# Nanobodies Are Potential Therapeutic Agents for the Ebola Virus Infection

**DOI:** 10.32607/actanaturae.11487

**Published:** 2021

**Authors:** I. B. Esmagambetov, D. V. Shcheblyakov, D. A. Egorova, O. L. Voronina, A. A. Derkaev, D. V. Voronina, O. Popova, E. I. Ryabova, D. N. Shcherbinin, E. I. Aksenova, A. N. Semenov, M. S. Kunda, N. N. Ryzhova, O. V. Zubkova, A. I. Tukhvatulin, D. Yu. Logunov, B. S. Naroditsky, S. V. Borisevich, A. L. Gintsburg

**Affiliations:** Federal State Budgetary Institution “National Research Centre for Epidemiology and Microbiology named after the Honorary Academician N. F. Gamaleya” of the Ministry of Health of the Russian Federation, Moscow, 123098 Russia; 48 Central Research Institute, Ministry of Defense, Sergiev Posad-6, 141306 Russia

**Keywords:** Ebola virus, nanobody, recombinant adenoviral vector, recombinant vesicular stomatitis virus

## Abstract

Ebola fever is an acute, highly contagious viral disease with a mortality rate that can reach 90%. There are currently no licensed therapeutic agents specific to Ebola in the world. Monoclonal antibodies (MAbs) with viral-neutralizing activity and high specificity to the Ebola virus glycoprotein (EBOV GP) are considered as highly effective potential antiviral drugs. Over the past decade, nanobodies (single-domain antibodies, non-canonical camelid antibodies) have found wide use in the diagnosis and treatment of various infectious and non-infectious diseases. In this study, a panel of nanobodies specifically binding to EBOV GP was obtained using recombinant human adenovirus 5, expressing GP (Ad5-GP) for alpaca (Vicugna pacos) immunization, for the first time. Based on specific activity assay results, affinity constants, and the virus-neutralizing activity against the recombinant vesicular stomatitis virus pseudotyped with EBOV GP (rVSV-GP), the most promising clone (aEv6) was selected. The aEv6 clone was then modified with the human IgG1 Fc fragment to improve its pharmacokinetic and immunologic properties. To assess the protective activity of the chimeric molecule aEv6–Fc, a lethal model of murine rVSV-GP infection was developed by using immunosuppression. The results obtained in lethal model mice have demonstrated the protective effect of aEv6–Fc. Thus, the nanobody and its modified derivative obtained in this study have shown potential protective value against Ebola virus.

## INTRODUCTION

Ebola virus, a member of the Filoviridae family, genus Ebolavirus, is a causative agent of hemorrhagic fever in humans and non-human primates [1]. The genus Ebolavirus consists of six species: Zaire ebolavirus (EBOV), Sudan ebolavirus (SUDV), Bundibugyo ebolavirus (BDBV), Tai Forest ebolavirus (TAFV), Reston ebolavirus (RESTV), and Bombali ebolavirus (BOMV) [2]. The mortality rate of the disease caused by the Ebola virus (EVD) can top 60–90% [3, 4]. Recent EVD outbreaks were reported in the Congo in 2018, Uganda in 2019, and Congo and Guinea in 2021 [5].

Monoclonal antibody cocktails specific to EBOV GP can provide complete protection against EVD in non-human primates; some of them (Zmapp, MAb114, REGN-EB3, and GamEMab) are currently undergoing clinical trials [6, 7, 8]. In our previous study [9], we immunized mice with Ad5-GP (component B of the GamEvac-Combi vaccine [10]) and managed to obtain two mouse monoclonal antibodies (2c8 and 6g3) that are protective against EVD.

In addition to monoclonal antibodies, the possibility of using nanobodies (non-canonical forms of camelid monoclonal antibodies) and their modified derivatives in the treatment of infectious diseases has been an area of interest for the past 20 years [11, 12]. However, there are only a few studies on the development of anti-EVD nanobody drugs [13, 14, 15]. The main advantages of nanobodies are a relatively straightforward technology of production and their ability to bind to hidden antigenic epitopes [13, 14, 15]. The disadvantages of nanobodies are associated with their rapid excretion by kidneys and the fact that the Fc fragment lacks an independent effector function. The Fc fragment of IgG requires some modification to improve the pharmacokinetic and effector properties of nanobodies [12] and increase their avidity due to molecule dimerization.

We have obtained a nanobody with protective activity against the recombinant vesicular stomatitis virus pseudotyped with the Ebola virus glycoprotein (rVSV-GP). For this, we used a technology that included the following steps: (1) immunization of alpaca with Ad5-GP, (2) generation of a panel of nanobodies specific to EBOV GP, (3) selection of a clone with optimal activity in vitro, (4) modification of the selected clone to improve its pharmacokinetic and immunological properties, and (5) in vivo assessment of the protective effect of the selected clone.

We selected and characterized the most promising nanobody: aEv6. The protective activity of a modified form of this clone (aEv6–Fc) was evaluated in a lethal model of rVSV-GP infection in immunosuppressive mice. Mice were injected with dexamethasone and cyclophosphamide to induce immunosuppression. This approach has been previously used to assess the activity of antiviral drugs and study the factors of Ebola virus pathogenesis [16, 17]. Our study established the ability of the aEv6–Fc antibody to protect mice from a lethal rVSV-GP infection, which may indicate its potential antiviral activity against the Ebola virus.

## EXPERIMENTAL PROCEDURES

**Viruses and antigens**

The following viruses were used in the study: Ad5- GP, a recombinant replication-defective adenovirus expressing the GP Zaire ebola virus gene (Ebola virus/H. sapiens-wt/SLE/2014/Makona-G3735.1 isolate; GenBank Accession No. KM233056) obtained as previously described [10, 18] and rVSV-GP, a recombinant vesicular stomatitis virus expressing the GP Zaire ebolavirus gene (Ebola virus/H. sapiens-wt/ SLE/2014/Makona-G3735.1 isolate, GenBank Accession No. KM233056) obtained as previously described [10].

The antigens used in the study are as follows: recombinant protein GP Zaire ebolavirus (H. sapiens-wt/GIN/2014/Kissidougou-C15 strain; Sino Biological, China, Cat No. 40442-V02H) and helper phage M13 Hyperphage M13 K07ΔpIII (Progen, Germany, Cat No. PRHYPE).

**Cell lines**

The following cell lines were used in the study: CHO-S cells (Thermo Fisher Scientific, USA, Cat No. R80007); Vero E6 (ATCC CRL 1586) and GMK-AH-1(D) (CVCL_L878) cells were received from the Russian collection of cell cultures of vertebrates (St. Petersburg, Russia, https://www.incras.ru/institut/ struktura/ckp/rossijskaja-kollekcija-kletochnyh-kultur/).

**Alpaca immunization, immune library construction, nanobody expression and purification**

A healthy four-year old male alpaca (Vicugna pacos) was used for immunization and blood sampling. The animal was provided by the Russian Alpaca Farm (Pokhodkino, Russia).

Triple injections of Ad5-GP (10^8^ PFU), without adjuvants, were administered intramuscularly at three-week intervals. The recombinant EBOV GP protein (200 μg) with an incomplete Freund’s adjuvant was injected intramuscularly three weeks after the last injection of Ad5-GP.

A total of 150 ml of blood was sampled five days after the last immunization; blood samples were collected in sterile vacuum tubes containing lithium heparin.

Isolation of mRNA, PCR, library construction, and specific screening were performed according to [19] using the recombinant EBOV GP protein as an antigen.

Expression and purification of the nanobodies were carried out as previously described [19].

**Next-Generation Sequencing of nanobody genes**

Nanobody gene amplicons were generated using specific primers [19] and purified by the MinElute PCR Purification Kit (QiaGen, Netherlands). Libraries were prepared according to the random priming protocol (Roche, Switzerland). Library sizing and quantitation were performed using the High Sensitivity DNA Kit (Agilent, USA). Sequencing was performed using the GS Junior Titanium Sequencing Kit and GS Junior + Series XL + Kit GS Junior + Series XL (Roche), according to the manufacturer’s instructions.

Amplicons were analyzed using 454 Sequencing System Software v. 3.0 and our own software.

**Determination of nanobody affinity constants**

The affinity constants of the nanobodies were determined using the recombinant EBOV GP protein by surface plasmon resonance (SPR) on a Biacore 3000 instrument (GE Healthcare Bio-Sciences AB, Sweden) as previously described [19].

**Production of the modified nanobody aEv6–Fc**

The nucleotide sequence of aEv6–Fc (synthesized at ZAO Evrogen, Russia) was cloned into the pShuttle-CMV plasmid (Stratagene, USA) to obtain the plasmid pShuttle-CMV-aEv6Fc.

CHO-S cells (Thermo Fisher Scientific, USA) were transiently transfected with plasmid pShuttle-CMV-aEv6Fc using the CHOgro Expression System (Mirus Bio, USA) according to the manufacturer’s instructions. Cells were cultured in Erlenmeyer flasks in a 5% CO_2_ and 80% humidity at 32°C and 125 rpm; the temperature was reduced to 32°C after 24 h, and the cells were cultured for up to 10 days. Starting from day 3, the following supplements were added: Cell boosts 7a (2%) and 7b (0.2%) (HyClone, USA) and 0.5% CHO Feed Bioreactor Supplement (Sigma, USA) once a day. After 10 days of cultivation, the cell culture medium was clarified by centrifugation at 5,000 g. The antibody was purified by affinity chromatography on the AKTA Start Protein Purification System (Cytiva, Sweden) using 1-ml MAbSelect SuRe columns (Cytiva, Sweden) according to the manufacturer’s instructions. Additional purification and buffer exchange were performed on a XK 26/100 column (Cytiva) using a Superdex 200 prep grade resin (Cytiva).

**Production of the control antibody MAb114**

The amino acid sequence published by Corti D. et al. was used to obtain the control antibody MAb114 [7]. Nucleotide sequences encoding the heavy and light chains of MAb114 were synthesized at ZAO Evrogen and cloned into the pShuttle-CMV plasmid (Stratagene). The resulting plasmids pShuttle-CMV-Mab114HC and pShuttle-CMV-Mab114LC were used to transfect CHO-S cells. Cell transfection and antibody purification were performed as described in the previous section.

**Indirect ELISA**

Indirect ELISA was carried out according to [9]. Anti-human IgG (Sigma, Cat No. A8667), Anti-Myc Tag (Abcam, Cat No. 1326), and anti-Llama IgG Heavy, and Light Chain (Bethyl, Cat No. A160-100P) antibodies conjugated to horseradish peroxidase (HRP) were used to detect nanobodies and antibodies (aEv6–Fc and MAb114) in alpaca serum.

**Virus neutralization assay**

Virus neutralization assay was performed as described in [9] using Vero E6 cells infected with the rVSV-GP virus as the model.

**Study of aEv6–Fc pharmacokinetics**

Three healthy rhesus macaques (Macaca mulatta) received from the Research Institute of Medical Primatology (Veseloye, Russia) were injected with 10 mg/kg aEv6–Fc intravenously with an infusion rate of 10 ml/h. Blood samples were collected before infusion and 1, 4, 8, 16, 24, 48, and 96 h and 7, 14, and 21 days after infusion. Indirect ELISA using various dilutions of aEv6–Fc as standards was used to assess the blood concentration of aEv6–Fc. Pharmacokinetic parameters were calculated using the Microsoft Excel and PKSolver software.

**Passive immunization and evaluation of protective activity**

Female BALB/c mice aged 4–6 weeks (weight, 18–20 g) were provided by the Pushchino Branch of the Institute of Bioorganic Chemistry, Russian Academy of Sciences (Pushchino, Russia). The mice were injected with dexamethasone (daily intraperitoneal injection of 10 mg/kg/day 10 days before virus injection) [17] and cyclophosphamide (single intraperitoneal injection of 150 mg/kg 5 days before virus injection) to induce immunosuppression. Recombinant VSV-GP was administered intravenously at a dose of 109 PFU/mouse; the aEv6–Fc antibody was injected intravenously at a dose of 50 mg/kg. The experimental animals were observed and weighed every day during the experiment. The results are presented as the animal survival rate (%) at different time points.

The experimental scheme is described in more detail in the Results section.

**Determination of rVSV-GP titers in the organs and tissues of infected mice**

Organs and tissues of infected mice were isolated under sterile conditions. Organ samples (20 mg each) were homogenized in 1 ml of DMEM. The suspension was clarified by centrifugation at 2,000 rpm for 10 min. The supernatant was collected to determine the virus-neutralizing titers of rVSV-GP.

**Statistical data analysis**

Data were analyzed using EXCEL 2010 and the STATISTICA v. 7.0 software. The Mann–Whitney U test and the Gehan–Wilcoxon test with a significance level of 0.05 were used to assess intergroup differences in the antibody titers and animal survival.

**Compliance with animal use regulations**

Experimental procedures were carried out in accordance with the Guide for the Care and Use of Laboratory Animals published by the National Institutes of Health (NIH Publication No. 85–23, revised in 1996) and the National Standard of the Russian Federation GOST R 53434–2009. All experiments were approved by the ethical committee of the N. F. Gamaleya National Research Center for Epidemiology and Microbiology (Minutes No. 27 of 2020). All individuals who worked with the animals and took care of them during the study received annual training in accordance with the IACUC requirements.

## RESULTS

**Production of a panel of nanobodies specific to EBOV GP**

**Fig. 1:**
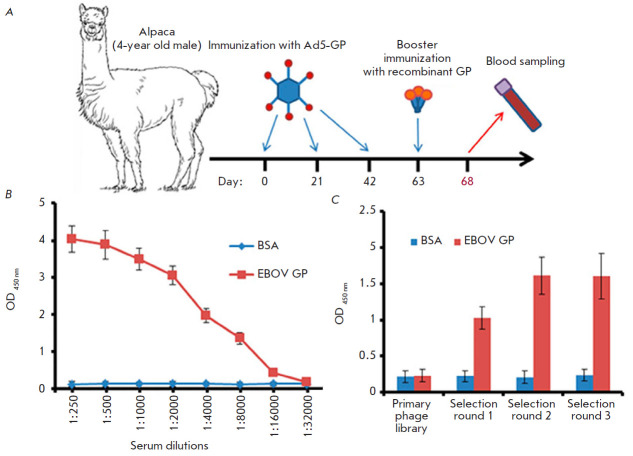
Schematic representation of alpaca (*Vicugna pacos*) immunization to obtain a library of nanobodies (*A*), anti-EBOV GP antibody titers in the serum of immunized alpaca (*B*), and polyclonal phage ELISA (*C*). (*B*) – High-binding Polystyrene Microtiter plates were coated with 100 μl (1 μg/ml) of EBOV GP (*H. sapiens-wt/GIN/2014/Kissidougou-C15*). On the next day, the wells were washed with 0.1% PBST five times and blocked with 5% non-fat skim milk in PBST. Different dilutions of the serum in PBST were added, and the samples were incubated at 37°C for 1 h. The wells were washed five times, and Anti-Llama IgG Heavy- and Light-Chain antibodies (Bethyl, USA, A160-100P) in blocking buffer (1 : 5,000) were added for incubation at 37°C for 1 h. The wells were washed five times, TMB was added, and the results were evaluated. (*C*) – High-binding Polystyrene Microtiter Plates were coated with 100 μl of EBOV GP (*H.sapiens-wt/GIN/2014/Kissidougou-C15*). On the next day, the wells were washed with 0.1% PBST five times and then blocked with 5% non-fat skim milk in PBST. A total of 1011 phages from each stage of the selection were added in PBST and incubated at 37°C for 1 h. The wells were washed five times to remove unbound phages, and HRP-conjugated Anti-M13 antibodies (Abcam, UK, B62-FE2) in blocking buffer (1 : 5,000) were added for incubation at 37°C for 1 h. The wells were washed five times, TMB was added, and the results were evaluated

A panel of nanobodies specific to EBOV GP was obtained by immunizing alpaca (V. pacos) with the recombinant Ad5-GP adenovirus and recombinant GP protein, according to the scheme presented in Fig. 1A.

On day 5 after booster immunization, 150 ml of alpaca blood was collected; serum was isolated to evaluate the titer of antibodies specific to EBOV GP to confirm the effectiveness of immunization. The titer of antibodies against EBOV-GP was determined at a dilution of 1 : 16,000, which indicates that the immunized animal has a high immune response (Fig. 1B).

Peripheral blood mononuclear cells (PBMCs) were isolated to create an immune library of nanobodies. Nucleotide sequences encoding variable fragments of nanobodies obtained from alpaca’s PBMCs were cloned into the phagemid vector pHEN1. Specific antibodies were selected by phage display as described in [19].

The selection results were analyzed using polyclonal phage ELISA (Fig. 1C). The library obtained after the second round of selection was used in further studies to prevent a decrease in nanobody diversity. Nucleotide sequences of nanobodies were identified by Next Generation Sequencing (NGS).

**Table 1:** NGS analysis of nanobody libraries

Clone	Read frequency, %		Amplification factor	CDR3 (amino acid sequence)
	Primary library	Library after selection		
aEv1	0.33	18.89	57.79024	NVQLGRFGILE
aEv2	0.31	7.81	24.89199	KSRRYGVDYW
aEv3	0.01	3.70	282.6006	AAVNSWAVYSLSRNYDY
aEv4	0.05	2.01	38.44366	AMRRGGVSYTYW
aEv5	0,01	3.71	283.5078	AVRSERYTRRYDH
aEv6	0.01	0.42	31.75288	YVDARYGALHTYRS
aEv7	0.08	1.60	20.41256	NAHYWSRD
aEv8	0.01	3.38	258.5591	KVTRGDFLGRRTDY
aEv9	0.01	0.20	14.96921	AARPGSYSRDARRYD
aEv10	0.03	2.08	79.609	NAQLSRSVLWGRY
aEv11	0.01	1.29	98.88753	QQKYAGRLY
aEv12	0.05	0.97	18.59811	AADRVLTSSSRNWDY
aEv13	0.01	1.54	117.9393	YARRRTYLAAY
aEv14	0.01	0.61	46.72209	AAGRSSMGLLDATDWRH
aEv15	0.01	0.85	65.3202	NSRGRHDWNRYN
aEv16	0.01	0.42	32.20649	AASPRTSMLVVGNVDH
aEv17	0.01	1.39	106.5989	NAQSHFFGSNY
aEv18	0.05	0.14	2.721675	AARPEYYSGTASYVSTSYDY
The remaining	98.954	48.99	0.494915	

**Fig. 2:**
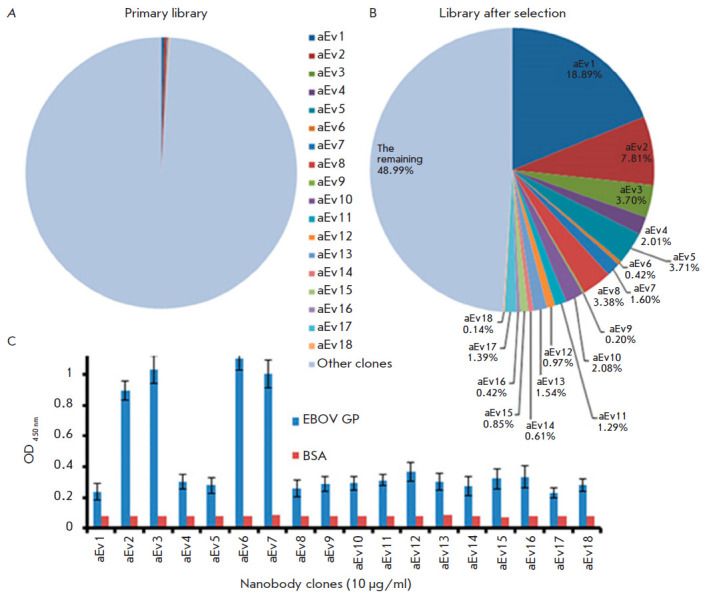
Comparative analysis of the percentage of nanobodies in the primary library before and after selection. (*A*) – percentage of nanobodies in the primary library. (*B*) – percentage of nanobodies in the library after selection. (*C*) – Screening of 18 nanobody clones by indirect ELISA. aEv1–18 – nanobody clones, EBOV GP – recombinant EBOV GP, BSA – bovine serum albumin (negative control)

A total of 18 clones (aE1–18) were identified (Table 1). The amplification factor of 16 clones after two rounds of selection exceeded 20, which was an indication of their specific accumulation through EBOV GP binding. A library analysis also showed that the selected clones constituted about 51% of all amplicons in the library after selection, while the percentage of these clones in the primary library was < 1% (Fig. 2 A,B).

As a result of indirect ELISA using EBOV GP, the clones aEv2, aEv3, aEv6, and aEv7 were selected as the most specific ones (Fig. 2C).

Wells were coated with 100 µl (1 µg/ml) of EBOV GP (H. sapiens-wt/GIN/2014/Kissidougou-C15). On the next day, the wells were washed with 0.1% PBST five times and blocked with 5% non-fat skim milk in PBST. Samples of the selected clones (10 µg/ml) were added to the wells and incubated in blocking buffer at 37°C for 1 h. The wells were washed five times, HRP-conjugated Anti-Myc Tag antibodies in blocking buffer (1 : 5,000) were added, with further incubation at 37°C for 1 h. The wells were washed five times, TMB was added, and the results were evaluated.

**Analysis of the selected clones by indirect ELISA, SPR, and virus neutralization assay**

**Fig. 3:**
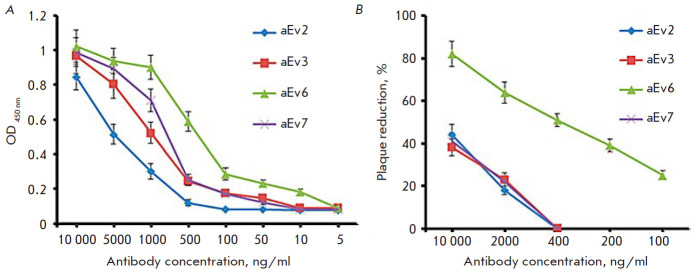
The activity of the selected nanobody clones evaluated by indirect ELISA (*A*) and the virus-neutralizing activity assessed using rVSV-GP (*B*). (*A*) – High-binding Polystyrene Microtiter plates were coated with 100 μl (1 μg/ml) of EBOV GP (*H. sapiens-wt/GIN/2014/Kissidougou-C15*). On the next day, the wells were washed with 0.1% PBST five times and blocked with 5% non-fat skim milk in PBST. Different dilutions of the nanobodies in blocking buffer were added to the plates and incubated at 37°C for 1 h. The wells were washed five times, and HRP-conjugated Anti-Myc Tag antibodies (Abcam, UK, ab1326) in blocking buffer (1 : 5,000) were added for incubation at 37°C for 1 h. The wells were washed five times, TMB was added, and the results were evaluated. (*B*) – Dilutions of rVSV-GP (*H. sapiens*-*wt/SLE/2014/Makona-G3735.1*) in buffer (10 mM Tris-HCl, pH 7.5; 1mM EDTA, 10% sucrose) were prepared. A mixture of equal volumes of the nanobodies and virus stocks was incubated at 37°C for 1 h and then transferred to Vero E6 cell monolayers. After cell incubation with the nanobody + virus complex at 37°C for 2 h, the cells were coated with agar. The plates were incubated in 5% CO_2_ atmosphere at 37°C for 48 h. The results were evaluated by counting the number of plaques under the microscope. The assay was performed in triplicate. The following formula was used to determine the plaque-forming units (PFU) per milliliter: PFU/ml = (mean PFU count/0.2 ml) × dilution factor

The immunological properties of the selected clones aEv2, aEv3, aEv6, and aEv7 were studied by ELISA, SPR (determination of affinity constants, KD), and virus neutralization assay in vitro; the main results are shown in Fig. 3.

Indirect ELISA showed the following optimal titers for the clones aEv2, aEv3, aEv7, and aEv6: ≥ 1 μg/ml, 500 ng/ ml, 500 ng/ ml, and 50 ng/ml, respectively (Fig. 3A). Clone aEv6 had the lowest KD value (1.87 × 10^-10^ M), followed by clones aEv3 and aEv7: 5.53 × 10^-8^ and 2.4 × 10^-8^ M, respectively. Clone aEv2 had the lowest K_D_ value: 7.13 × 10^-7^ M. As shown in Fig. 3B, aEv2, aEv3, and aEv7 did not exhibit any significant neutralizing activity against rVSV-GP, while aEv6 showed 50% neutralizing activity starting at a concentration of 400 ng/ml. Based on the obtained results, aEv6, which had the highest affinity for EBOV GP and the highest virus-neutralizing potential against rVSV-GP, was selected for further study.

**Production of the aEv6 clone modified with the Fc fragment of human IgG1 and analysis of its properties**

**Fig. 4:**
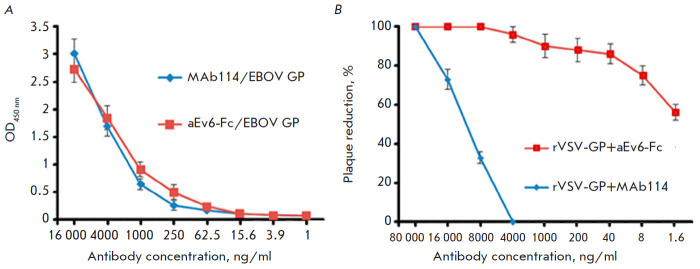
Comparison of the specific activity of aEv6–Fc and MAb114 against EBOV GP of the *H.sapiens-wt/GIN/2014/ Kissidougou-C15 *strain (*A*) and the virus-neutralizing activity of aEv6–Fc and MAb114 against rVSV-GP (*B*). (*A*) – High-binding Polystyrene Microtiter plates were coated with 100 μl (1 μg/ml) of EBOV GP (*H. sapiens-wt/GIN/2014/ Kissidougou-C15*). On the next day, the wells were washed with 0.1% PBST five times and blocked with 5% non-fat skim milk in PBST. Different dilutions of aEv6–Fc and MAb114 in blocking buffer were added to plates and incubated at 37°C for 1 h. The wells were washed five times, HRP-conjugated Anti-Human IgG antibodies (Sigma, USA) in blocking buffer (1 : 5,000) were added for incubation at 37°C for 1 h. The wells were washed five times, TMB was added, and the results were evaluated. (*B*) – Dilutions of rVSV-GP in buffer (10 mM Tris-HCl, pH 7.5; 1mM EDTA, 10% sucrose) were prepared. A mixture of equal volumes of the antibodies and virus stocks was incubated at 37°C for 1 h and then transferred to Vero E6 cell monolayers. After cell incubation with the antibody + virus complex at 37°C for 2 h, the cells were coated with agar. The plates were incubated in 5% CO_2_ atmosphere at 37°C for 48 h. The results were evaluated by counting the number of plaques under the microscope. The assay was performed in triplicate. The following formula was used to determine the plaque-forming units (PFU) per milliliter: PFU/ml = (mean PFU count/0.2 ml) × dilution factor

The selected aEv6 clone was modified with a human IgG1 Fc fragment to improve its immunological and pharmacokinetic properties. The resulting antibody aEv6–Fc showed specific activity against EBOV GP of the H. sapiens-wt/GIN/2014/Kissidougou-C15 strain, which was similar to the activity of the MAb114 used as a control (Fig. 4A).

Then, the virus was neutralized using rVSV-GP to compare the antibodies aEv6–Fc and MAb114. The aEv6–Fc antibody had a significantly higher neutralizing activity than MAb114 (Fig. 4B).

The study of the pharmacokinetic properties of aEv6–Fc in rhesus macaques showed that the average circulation time of the antibodies in the blood after injection is at least 7 days (data not shown), which is much higher than that of unmodified low-molecular-weight nanobodies [20, 21].

**Evaluation of aEv6–Fc protective activity in a lethal model of murine rVSV-GP infection**

At the last stage, the protective effect of aEv6–Fc was evaluated in a lethal rVSV-GP infection model in mice. While the non-human primate model is the most representative, financial and ethical considerations drive the development of new, small animal models [22]. In addition, all studies involving filoviruses require a biosafety level 4 [22]. All of this makes it reasonable to replace the natural Ebola virus with a recombinant analogue that is safer for humans. An example of such an analogue is the recombinant vesicular stomatitis virus pseudotyped with the EBOV GP protein (rVSV-GP). Since rVSV-GP is non-pathogenic in mice, the latter were injected with dexamethasone and cyclophosphamide prior to virus administration to induce immunosuppression [17]. The experiment was performed as follows: five groups of six mice each were selected. All groups were subjected to immunosuppressive therapy for 10 days. After that, the mice of the first group received virus injections, while other groups received virus+antibody injections.

**Fig. 5:**
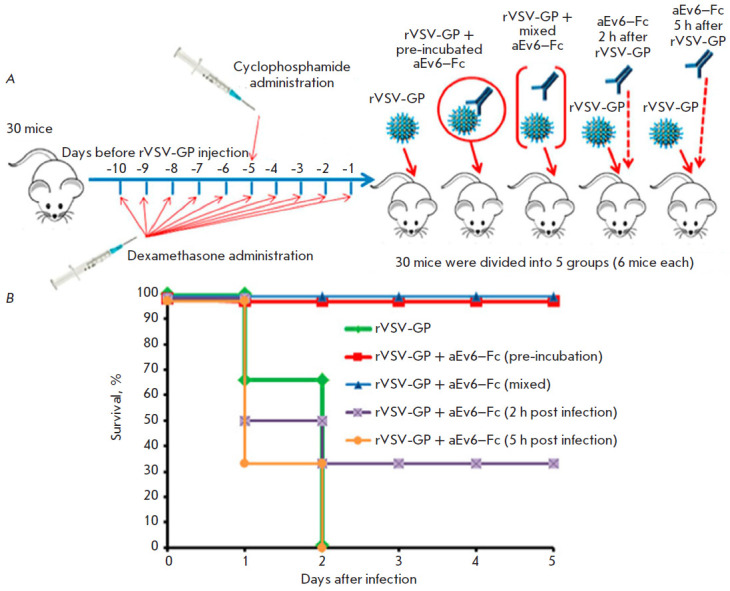
Protective activity of the aEv6–Fc antibody. Experimental scheme (*A*) and results of the experiment (*B*). (*A*) – rVSV-GP – recombinant live-attenuated vesicular stomatitis virus expressing EBOV GP (*H. sapiens-wt/SLE/2014/Makona-G3735.1*); aEv6–Fc – nanobody clone fused to the human IgG Fc fragment. (*B*) – rVSV-GP – mice challenged with rVSV-GP (109 PFU/mice); rVSV-GP+aEv6–Fc (pre-incubation) – mice receiving rVSV-GP (109 PFU/mice) that has been previously mixed and incubated with 300 μl of aEv6–Fc (3 mg/ml); rVSV-GP+aEv6–Fc (mixed) – mice injected with rVSV-GP (109 PFU/mice) that has been previously mixed with 300 μl of aEv6–Fc (3 mg/ml); rVSV-GP+aEv6–Fc (2 h post infection) – mice challenged with rVSV-GP (109 PFU/mice) and treated with 300 μl of aEv6–Fc (3 mg/ml) after 2 h; rVSV-GP+aEv6–Fc (5 h post infection) – mice receiving rVSV-GP (109 PFU/mice) and treated with 300 μl of aEv6–Fc (3 mg/ml) after 5 h

On day 11, mice were injected intravenously with 109 PFU of rVSV-GP in both the absence and presence of aEv6–Fc either pre-incubated with the virus at 37°C for 1 h, mixed with the virus immediately before injection, administered intravenously 2 h after infection, or administered intravenously 5 h after infection. The experimental scheme is presented in Fig. 5A.

The mice were observed for 5 days after infection. The control mice that did not receive aEv6–Fc died on day 2 after infection. Administration of aEv6–Fc 5 h after infection also failed to either prevent or delay animal death. Introduction of antibodies 2 h after infection resulted in the survival of two out of six mice. Pre-incubation and mixing of aEv6–Fc with rVSV-GP fully protected the animals. The experimental results are shown in Fig. 5B.

**Table 2:** Titers of rVSV-GP in the organs of infected mice

Group	Day after injection	Average rVSV-GP titers (two mice), PFU/20 μg of organ					
		blood	brain	liver	kidney	spleen	intestine
Intact immunosuppressed mice	1	-	-	-	-	-	-
	2	-	-	-	-	-	-
Immunosuppressed mice infected with rVSV-GP	1	4.44 × 10^5^	–	6.67 × 10^4^	4.14 × 10^4^	3.65 × 10^4^	–
	2	1.7 × 10^7^	–	1.27 × 10^5^	2.44 × 10^4^	3.44 × 10^4^	–
Immunosuppressed mice receiving rVSV-GP+aEv6–Fc	1	-	-	-	-	-	-
	2	-	-	-	-	-	-

For a more detailed assessment of the protective properties of aEv6–Fc against rVSV-GP in a mouse infection model, PFU was determined in the blood and organs of infected mice. Mice were divided into three groups of four animals each. The first group of immunosuppressed mice remained intact. The mice of the second group were infected with rVSV-GP. The third group was challenged with rVSV-GP that had been previously neutralized with aEv6–Fc (900 μg). The presence of rVSV-GP in the brain, liver, kidney, spleen, intestine, and blood of infected mice was determined using VeroE6 cells 1 and 2 days after. The experiment results are shown in Table 2. No signs of viral presence were noted in the tissues and organs of immunosuppressed mice free of the rVSV-GP infection (negative control). The virus was detected in the blood, liver, kidneys, and spleen of the animals in the second group (immunosuppressed mice infected with rVSV-GP) on the first day after its administration. Significantly higher titers of rVSV-GP were observed in the blood and liver on day 2, while no virus was detected in the organs and tissues of mice receiving rVSV-GP + aEv6–Fc injections (group 3).

Thus, experimental data confirm that aEv6–Fc has a virus-neutralizing and protective effect against a lethal rVSV-GP infection in immunosuppressed mice.

## DISCUSSION

In this study, the possibility of producing a nanobody fused to the Fc fragment of human IgG1, which has a neutralizing and protective activity against the vesicular stomatitis virus pseudotyped with the GP protein of the Ebola virus, was shown for the first time. In addition, a panel of nanobodies specific to EBOV GP was obtained for the first time by immunizing alpaca with the recombinant adenovirus Ad5-GP. This immunization strategy has previously been used successfully to obtain monoclonal antibodies with a protective activity against the Ebola virus [9]. Thus, four nanobody clones (aEv2, aEv3, aEv6, and aEv7) were obtained; their characteristics are presented in Table 3.

**Table 3:** Immunogenic characteristics of the clones aEv2, aEv3, aEv6, and aEv7

Clone	Titers for EBOV GP, ng/ml	Affinity constant (KD) for EBOV GP, M	Virus-neutralizing activity against rVSV-GP (PRNT50)
aEv2	≥ 1,000	7.13 × 10^-7^	No virus-neutralizing activity
aEv3	≥ 500	5.53 × 10^-8^	≥ 400 ng/ml
aEv6	≥ 50	1.87 × 10^-10^	No virus-neutralizing activity
aEv7	≥ 500	2.4 × 10^-8^	No virus-neutralizing activity

Our analysis of the data presented in Table 3 allows us to conclude that the results of three independent experiments completely correlated with each other. Based on the data we obtained, the aEv6 clone showed both the highest affinity for EBOV GP and virus-neutralizing activity against rVSV-GP. Therefore, this clone was selected for further study.

The aEv6 clone was further modified with the human IgG1 Fc fragment, resulting in a 40–45 kDa nanobody; the Fc fragment dimerizes the molecule and enables its interaction with the Fc receptors on the cell surface [21]. This modification increased the circulation duration of the aEv6–Fc antibody in the blood of non-human primates for up to 7 days, considering that the circulation of primary nanobodies usually lasts only several hours [20, 23, 24]. We used non-human primates, because their immune system and Fc receptors are highly homologous to those in humans. Improving antibody pharmacokinetics is an important aspect that can significantly reduce the dose and number of drug injections in the treatment of viral diseases. ELISA showed that the modified antibody aEv6–Fc had specific activity similar to that of MAb114 (Fig. 4A), which has a protective activity against the Ebola virus [25], and a stronger virus-neutralizing activity than both MAb114 and an unmodified antibody lacking the Fc fragment (Fig. 4B).

The last stage of the study was the assessment of the protective activity of aEv6–Fc in a mouse model of lethal infection with the vesicular stomatitis virus pseudotyped with EBOV GP. We developed this model to avoid the need for a wild-type Ebola virus and non-human primates in the study (due to the high cost and ethical considerations [22]). The drug dose (50 mg/kg) was selected based on published data [25]. Our experiments have shown that aEv6–Fc completely protects mice from infection when either pre-incubated or mixed with the virus prior to injection and, in our case, had a 30%-protection level when administered no later than 2 h after infection with the virus. Thus, aEv6–Fc can be used as a protective agent for both prevention and treatment immediately after suspected contact with the pathogen. A detailed analysis of rVSV-GP accumulation in the organs and tissues of the infected mice revealed the highest virus titer in the blood, liver, kidneys, and spleen, while no rVSV-GP was found in the brain and intestine. The obtained results may have to do with the pseudotyping of the vesicular stomatitis virus with EBOV GP, which apparently alters the tropism of the virus. Changes in rVSV tropism, in turn, may explain the virus accumulation in the kidneys, spleen, and especially in the liver and blood, which apparently causes multiple organ failure and animal death on the second day of infection. It is important to note that no virus was found in the organs and tissues of mice infected with rVSV-GP pre-incubated with aEv6–Fc, which once again confirms the virus-neutralizing and protective capacity of the antibody.

## CONCLUSION

In this study, the possibility of producing nanobodies and modified derivatives from them specific to the Ebola virus surface glycoprotein and exhibiting strong antiviral activity in a lethal model of mice infected with a pseudotyped vesicular stomatitis virus was shown for the first time.

## Abbreviations

EVD: Ebola virus disease
EBOV GP: Ebola virus glycoprotein
MAb: monoclonal antibody
PFU: plaque-forming unit
ELISA: enzyme-linked immunosorbent assay
TMB: 3,3’,5,5’-tetramethylbenzidine
PBST: phosphate buffered saline with polysorbate 20
HRP: horseradish peroxidase
PBMC: peripheral blood mononuclear cell
PRNT: plaque reduction neutralization test
PRNT50: 50% neutralization titer
